# Supplementary material for: Construction of a two-dimensional artificial antioxidase for nanocatalytic rheumatoid arthritis treatment
Source: Nat Commun. 2022 Apr 13;13:1988. doi: 10.1038/s41467-022-29735-1 (PMC9008001; doi:10.1038/s41467-022-29735-1)
Supplement: Supplementary file 1 — Supplementary Information [file 41467_2022_29735_MOESM1_ESM.pdf]

Supplementary Information

Construction of a Two-Dimensional Artificial Antioxidase for Nanocatalytic Rheumatoid Arthritis Treatment

*Bowen Yang,[1,2] Heliang Yao,[1] Jiacai Yang,[1,2] Chang Chen,[1,2] and Jianlin Shi[1,3]**

[1] State Key Laboratory of High Performance Ceramics and Superfine Microstructure, Shanghai Institute of Ceramics, Chinese Academy of Sciences; Research Unit of Nanocatalytic Medicine in Specific Therapy for Serious Disease, Chinese Academy of Medical Sciences (2021RU012), Shanghai, 200050, P. R. China;

[2] Center of Materials Science and Optoelectronics Engineering, University of Chinese Academy of Sciences, Beijing, 100049, P. R. China;

[3] Tenth People's Hospital and Shanghai Frontiers Science Center of Nanocatalytic Medicine, School of Medicine, Tongji University, Shanghai, 200092, P. R. China.

E-mail: jlshi@mail.sic.ac.cn

Supplementary Figures

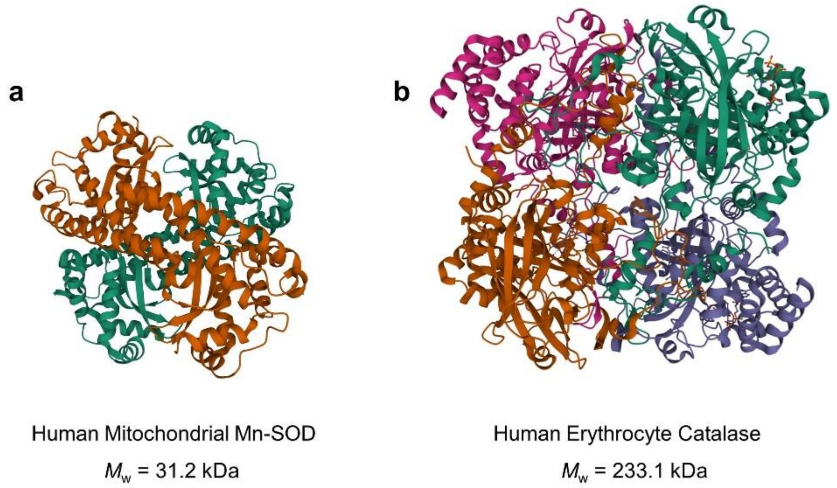

Supplementary Figure 1. 3D structures of human mitochondrial Mn-SOD (PDB: 1N0J) and human erythrocyte catalase (PDB: 1DGF), as well as their molecular weights.[1,2]

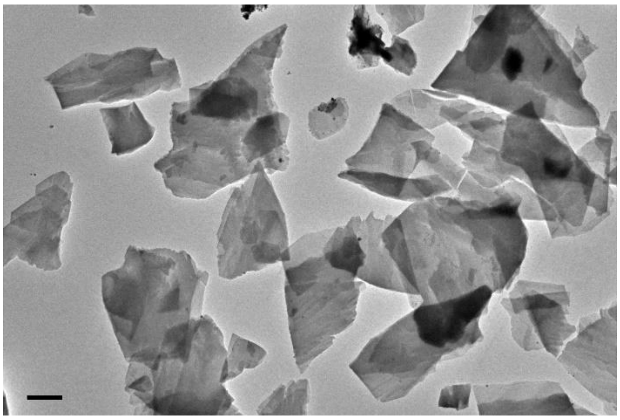

Supplementary Figure 2. TEM image of large ZMTP sheets synthesized from a solvothermal reaction. Scale bar, 2 μm. A representative image of three replicates is shown.

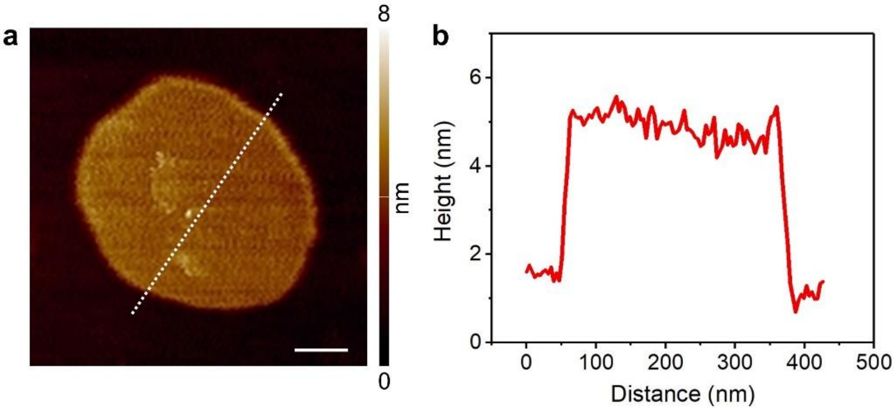

Supplementary Figure 3. AFM image of a single ZMTP nanosheet **(a)**, as well as corresponding thickness measurement **(b)**. Scale bar, 100 nm. A representative image of three replicates is shown. Source data are provided as a Source Data file.

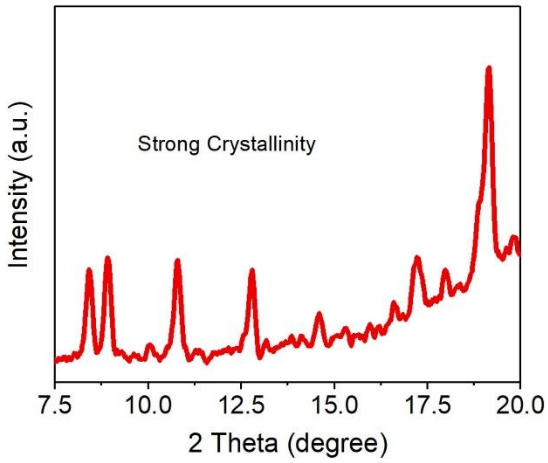

Supplementary Figure 4. XRD pattern of ZMTP nanosheets. Source data are provided as a Source Data file.

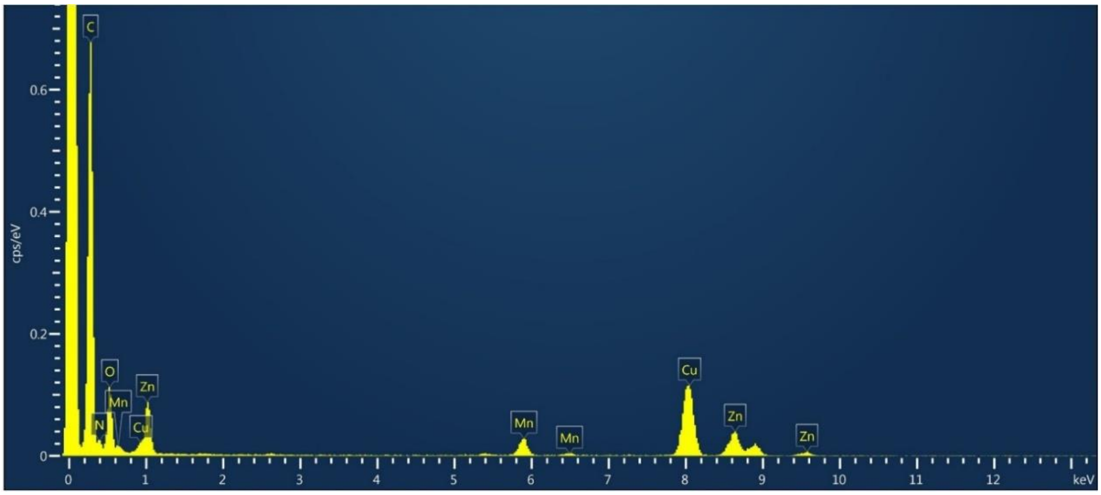

Supplementary Figure 5. EDS pattern of ZMTP sample collected on a carbon film of a cooper grid.

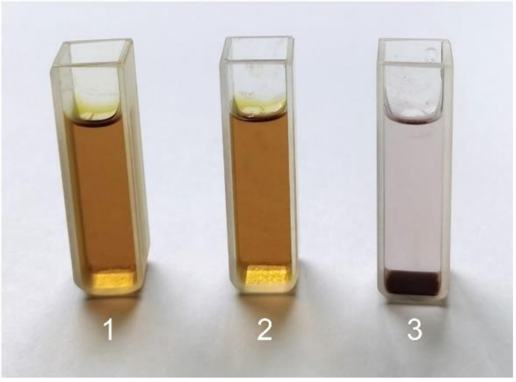

Supplementary Figure 6. Digital photos of ZMTP nanosheets dispersed in deionized water (1) and PBS (2) for 12 h. The nanosheets also underwent 8 rounds of ethanol washing, which would aggregate in PBS (3), as a consequence of the loss of the surfactant PVP.

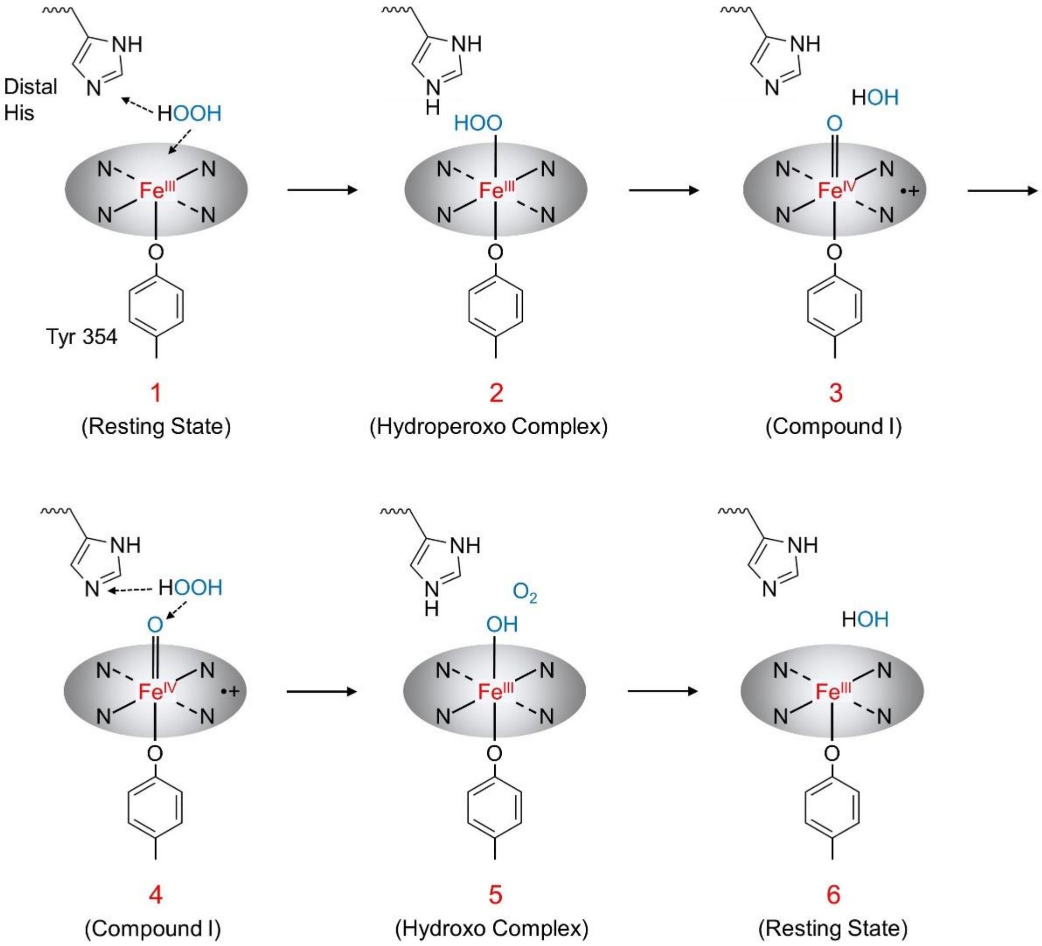

Supplementary Figure 7. Detailed reaction process for the H_2O_2 decomposition on the Fe center of catalase.[3]

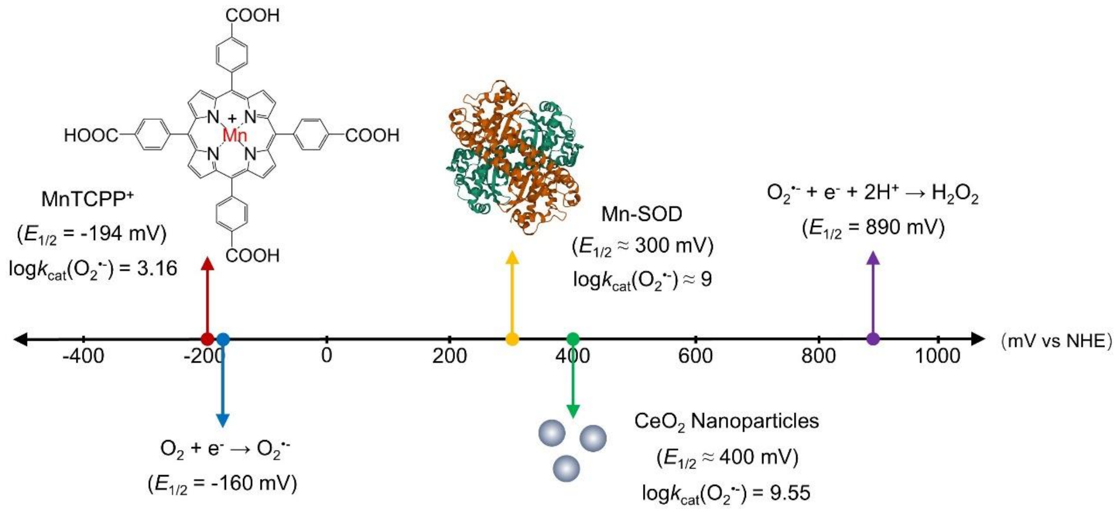

Supplementary Figure 8. Half-wave redox potentials ($E_{1/2}$, vs NHE) for oxidation and reduction reactions of $O_2^{\bullet-}$, as well as for various catalysts with SOD-enzymatic or mimicking activities. Here $E_{1/2}$ of $MnTCPP^+$ is for the metal-centered reduction potential of Mn^{III}/Mn^{II} redox couple, which is too negative to enable the first step of $O_2^{\bullet-}$ dismutation ($O_2^{\bullet-}$ oxidation).[4]

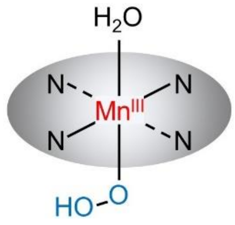

Supplementary Figure 9. Stereoscopic side view for the transient state of Mn-N_4 center of ZMTP nanosheets during the first step of catalytic H_2O_2 decomposition.[5] The deprotonation of H_2O_2 enables the formation of one hydroperoxide anion (HOO^-), which can replace the axial hydroxo ligand and coordinates with Mn center axially.

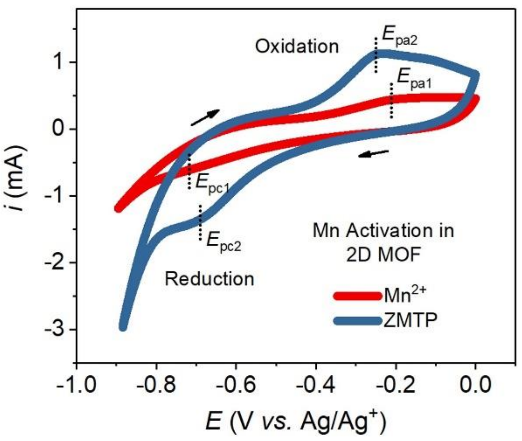

Supplementary Figure 10. CV curves of Mn^{2+} and ZMTP in electrolyte solutions investigating the redox responses. E_{pa1} and E_{pa2} correspond to the anodic peak potentials of Mn^{2+} and ZMTP in electrolyte solutions, respectively, while E_{pc1} and E_{pc2} correspond to the cathodic peak potentials of Mn^{2+} and ZMTP in electrolyte solutions, respectively. Source data are provided as a Source Data file.

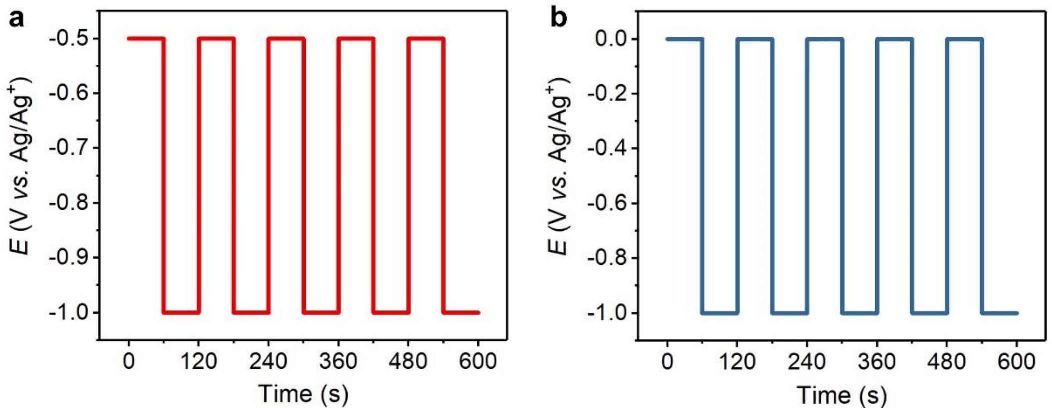

Supplementary Figure 11. (a) Five successive periods of the action and transition of dual step potentials (high potential: -0.5 V; low potential: -1.0 V) during CA measurement in Figure 3c. **(b)** Five successive periods of the action and transition of dual step potentials (high potential: 0 V; low potential: -1.0 V) during CA measurement in Figure 3d. Source data are provided as a Source Data file.

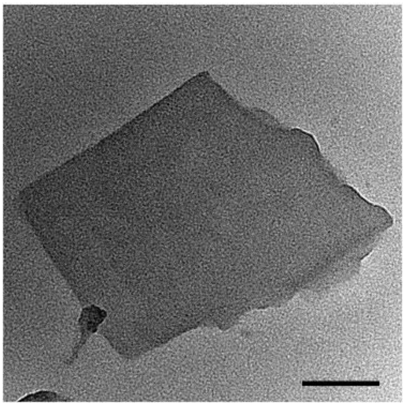

Supplementary Figure 12. TEM image of ZMTP nanosheet after CA measurement in Figure 3d. Scale bar, 200 nm. A representative image of three replicates is shown.

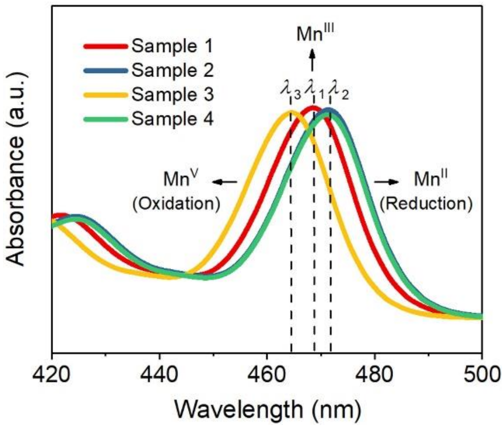

Supplementary Figure 13. UV-vis spectra of different samples revealing the generations of Mn^{II} and Mn^{V} species after reduction and oxidation reactions of ZMTP nanosheets in CA measurement. Sample 1 to 4 were extracted from the ZMTP nanosheet-containing electrolyte solution respectively at the end of the former half period of the first period during the CA measurement in Figure 3c, the end of the first complete period during the CA measurement in Figure 3c, the end of the former half period of the first period during the CA measurement in Figure 3d, as well as the end of the first complete period during the CA measurement in Figure 3d. Source data are provided as a Source Data file.

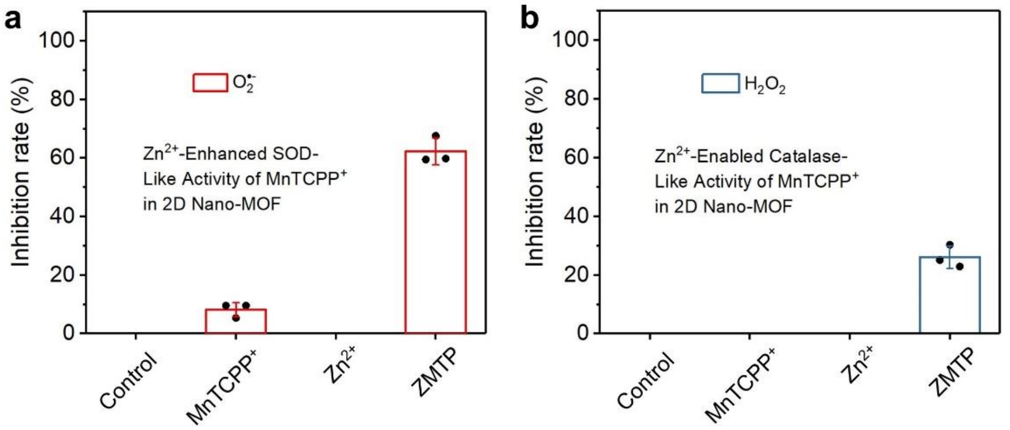

Supplementary Figure 14. Inhibition rates of $O_2^{\bullet-}$ **(a)** and H_2O_2 **(b)** in different groups in 2 h of reaction. Data are expressed as means ± SD (*N* = 3 independent experiments). Source data are provided as a Source Data file.

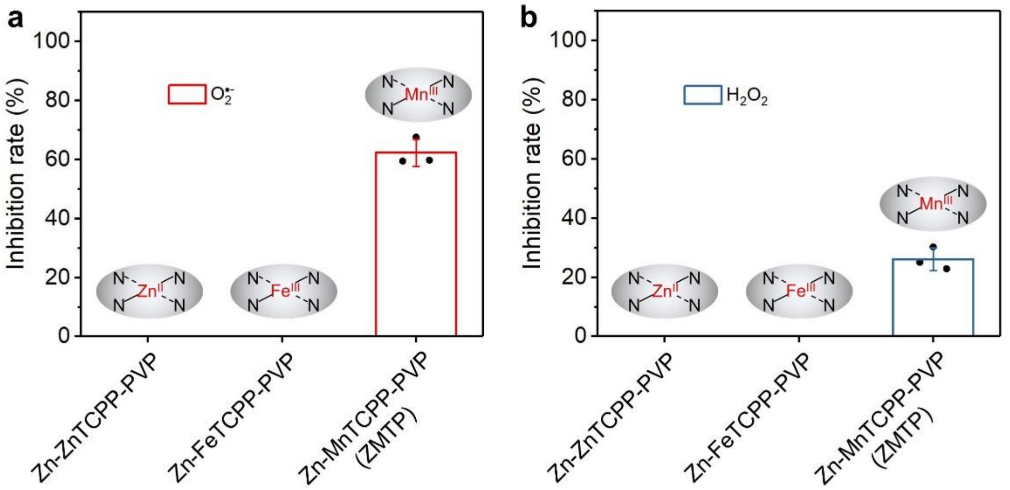

Supplementary Figure 15. Inhibition rates of $O_2^{\bullet-}$ **(a)** and H_2O_2 **(b)** in groups of Zn-ZnTCPP-PVP, Zn-FeTCPP-PVP and ZMTP in 2 h of reaction. Data are expressed as means ± SD (*N* = 3 independent experiments). Zn-FeTCPP-PVP can be considered as a peroxidase mimic. Source data are provided as a Source Data file.

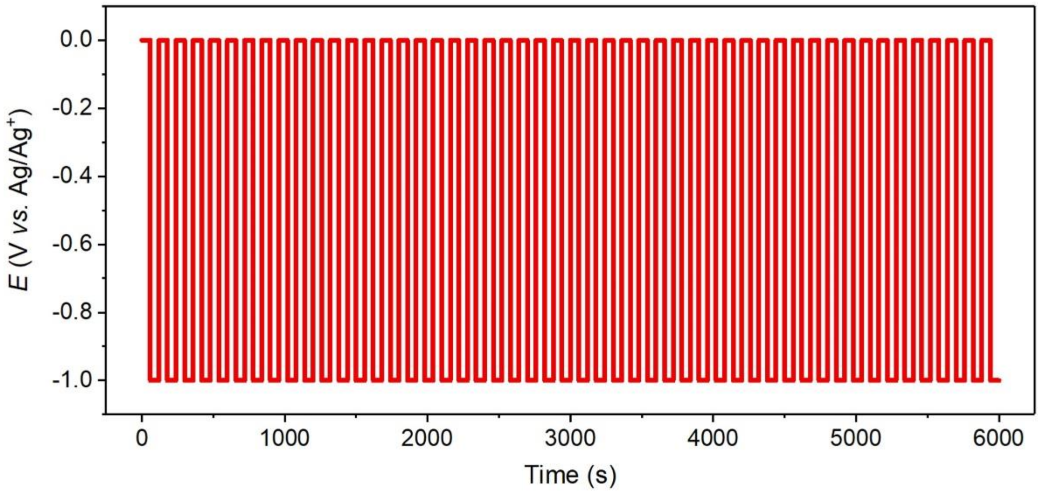

Supplementary Figure 16. Fifty cycles of dual step potential transitions in Figure 3f (high potential: 0 V; low potential: -1.0 V). Source data are provided as a Source Data file.

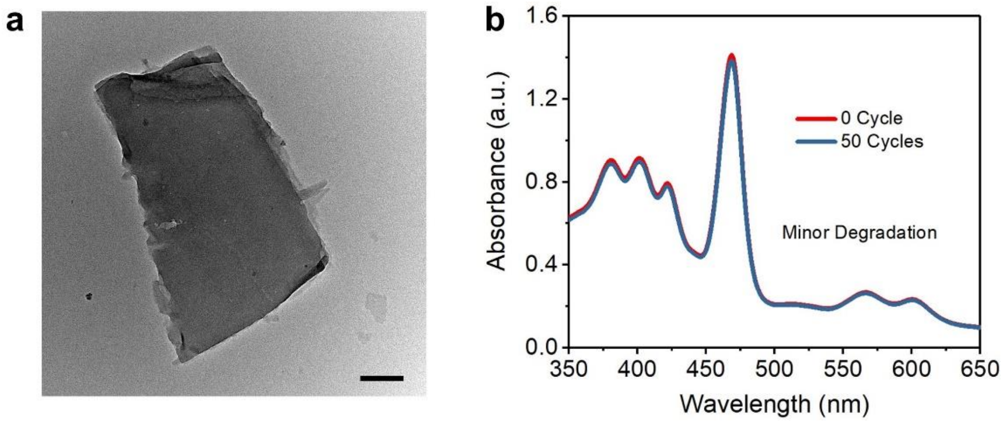

Supplementary Figure 17. (a) TEM image of a representative ZMTP nanosheet after 50 cycles of dual step potential transitions. Scale bar, 50 nm. A representative image of three replicates is shown. **(b)** UV-vis spectra of ZMTP nanosheets before and after 50 cycles of dual step potential transitions. Source data are provided as a Source Data file.

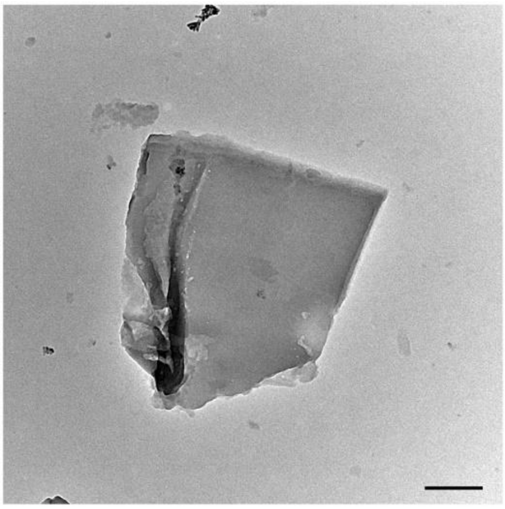

Supplementary Figure 18. TEM image of a representative ZMTP nanosheet after dispersing in mild acidic PBS for 12 h. Scale bar, 50 nm. A representative image of three replicates is shown.

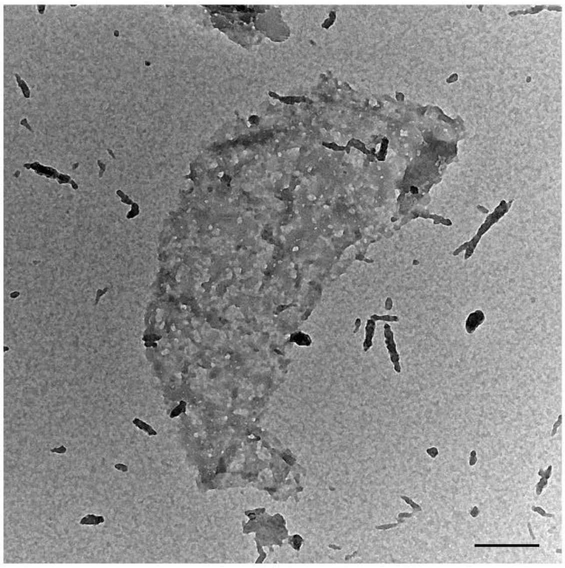

Supplementary Figure 19. TEM image of a representative ZMTP nanosheet after dispersing in mild acidic PBS for 24 h. Scale bar, 50 nm. A representative image of three replicates is shown.

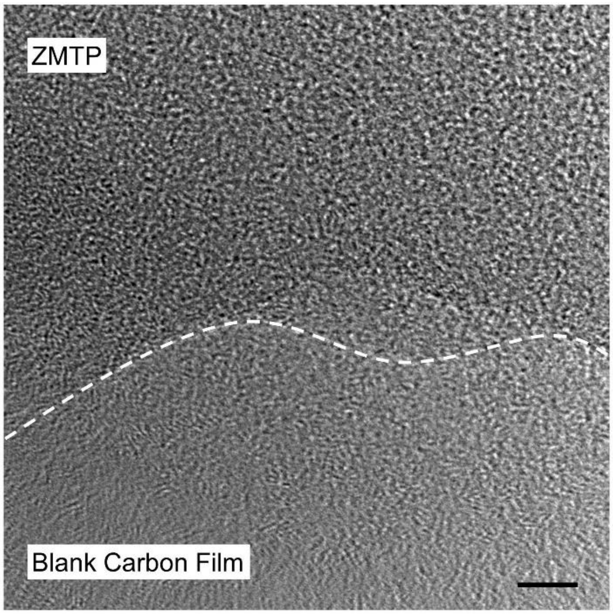

Supplementary Figure 20. High-resolution TEM image of ZMTP nanosheet after dispersing in mild acidic PBS for 24 h, indicating significantly lowered crystallinity during degradation. Scale bar, 5 nm. A representative image of three replicates is shown.

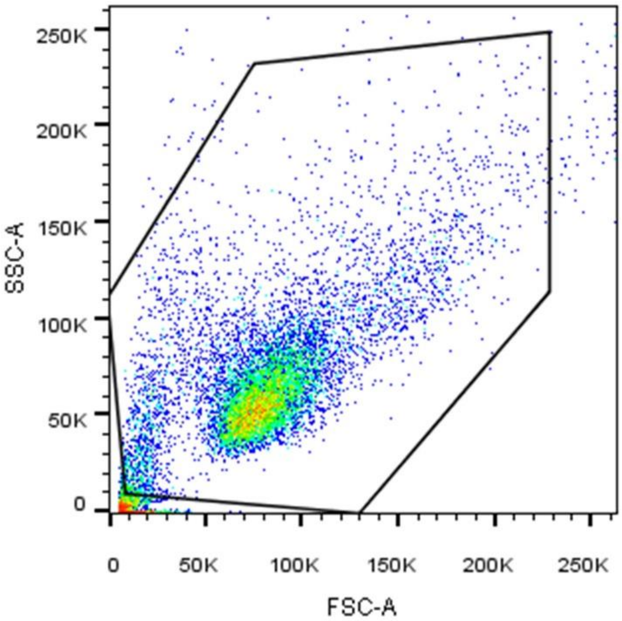

Supplementary Figure 21. Gating strategy to sort Raw264.7 cells for flow cytometric analysis in Figure 5d.

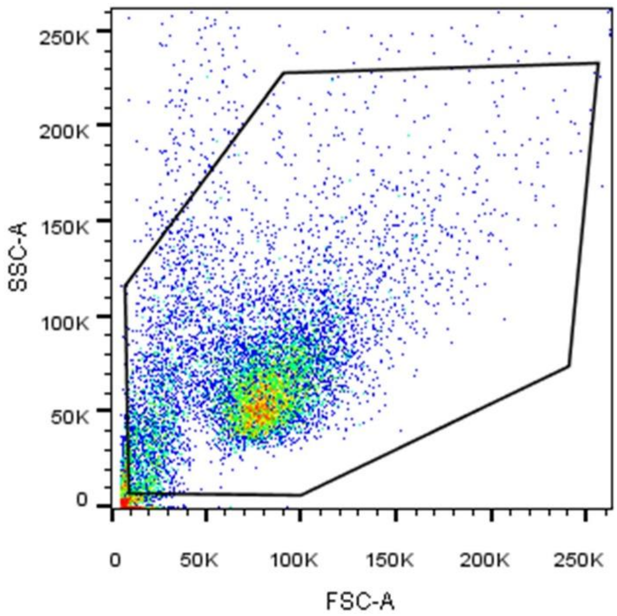

Supplementary Figure 22. Gating strategy to sort mBMSCs for flow cytometric analysis in Figure 6d.

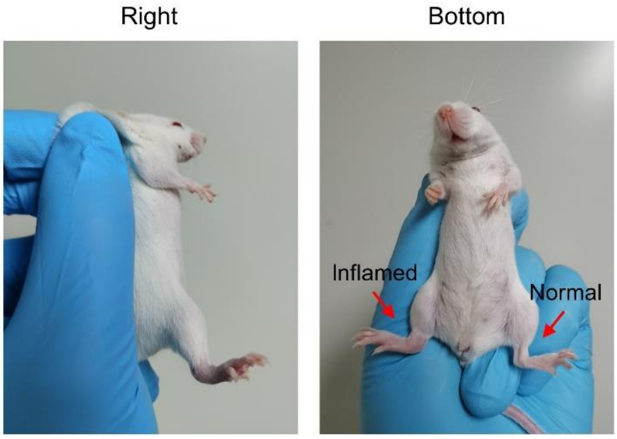

Supplementary Figure 23. Digital photos of mice after injection of complete Freund's adjuvant at right hind ankle joints for 15 days.

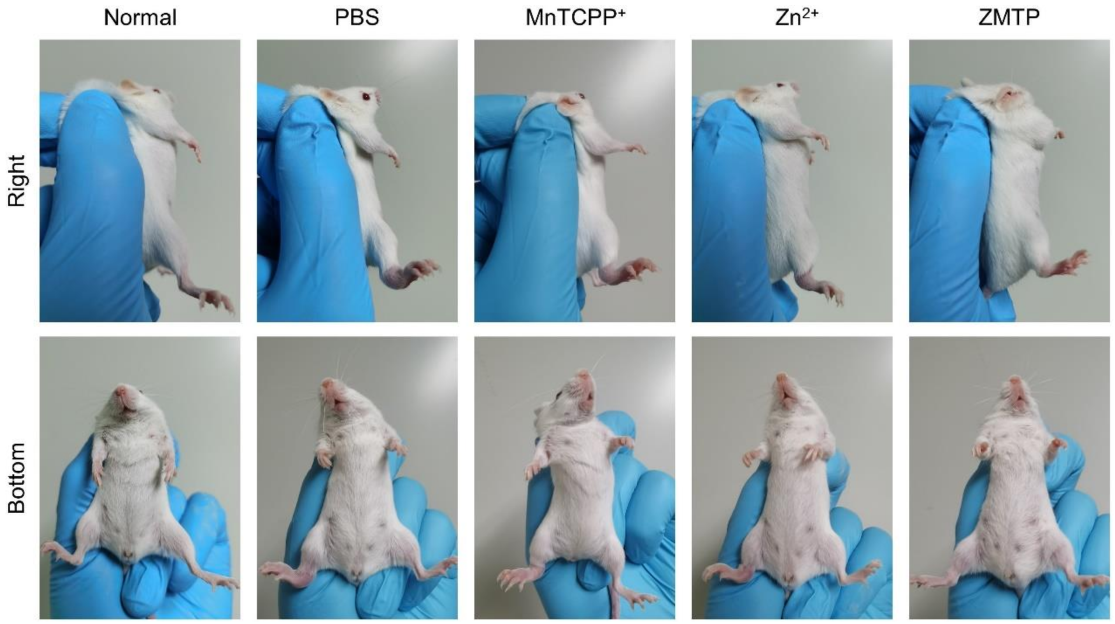

Supplementary Figure 24. Representative digital photos of mice in different groups on day 36.

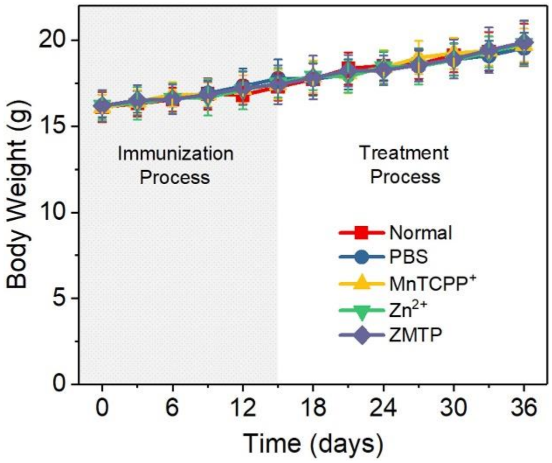

Supplementary Figure 25. Body weight of mice in different groups during a total observation period of 36 days. Data are expressed as means ± SD ($N = 5$ biologically independent animals). Source data are provided as a Source Data file.

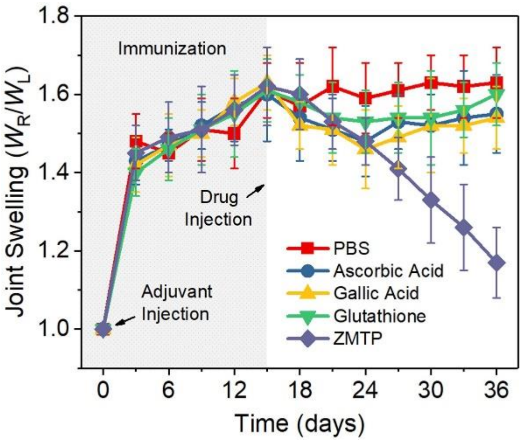

Supplementary Figure 26. Comparison of the therapeutic potencies between conventional antioxidants (ascorbic acid, gallic acid and reduced glutathione) and that of ZMTP. The data for arthritic mice in PBS and ZMTP groups are obtained from Figure 7a. Data are expressed as means ± SD (N = 5 biologically independent animals). Source data are provided as a Source Data file.

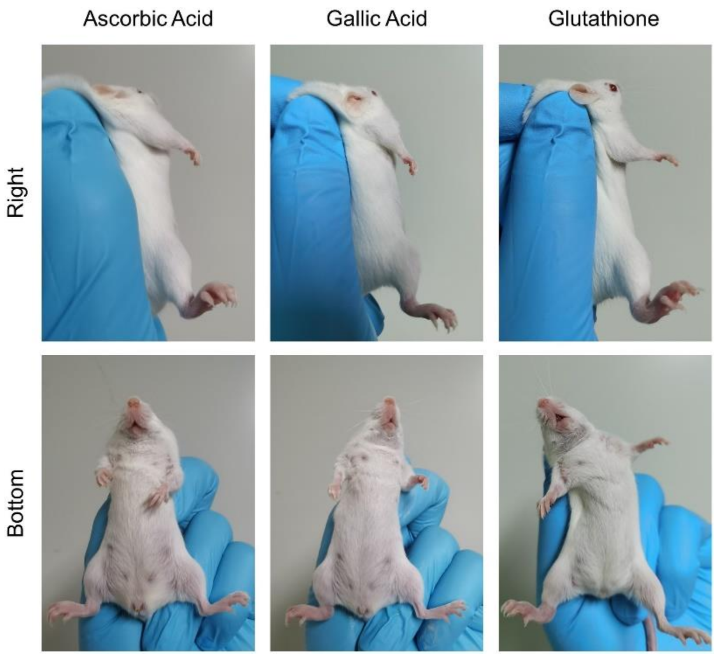

Supplementary Figure 27. Digital photos of arthritic mice after treatment with ascorbic acid, gallic acid or glutathione (reduced) on day 36, representative of five biological replicates from each group.

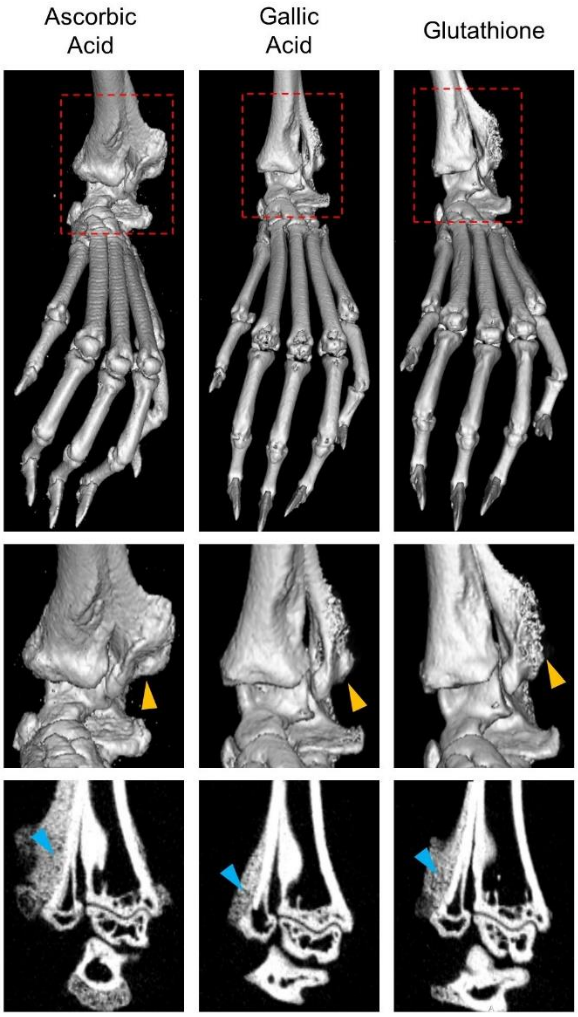

Supplementary Figure 28. 3D-reconstructed micro-CT images of right hind ankle joints of arthritic mice after treatment with ascorbic acid, gallic acid or glutathione (reduced) on day 36, as well as original micro-CT images of trabecular. A representative image of five replicates from each group is shown. Yellow and blue triangle marks reveal abnormal bone structures due to inflammation.

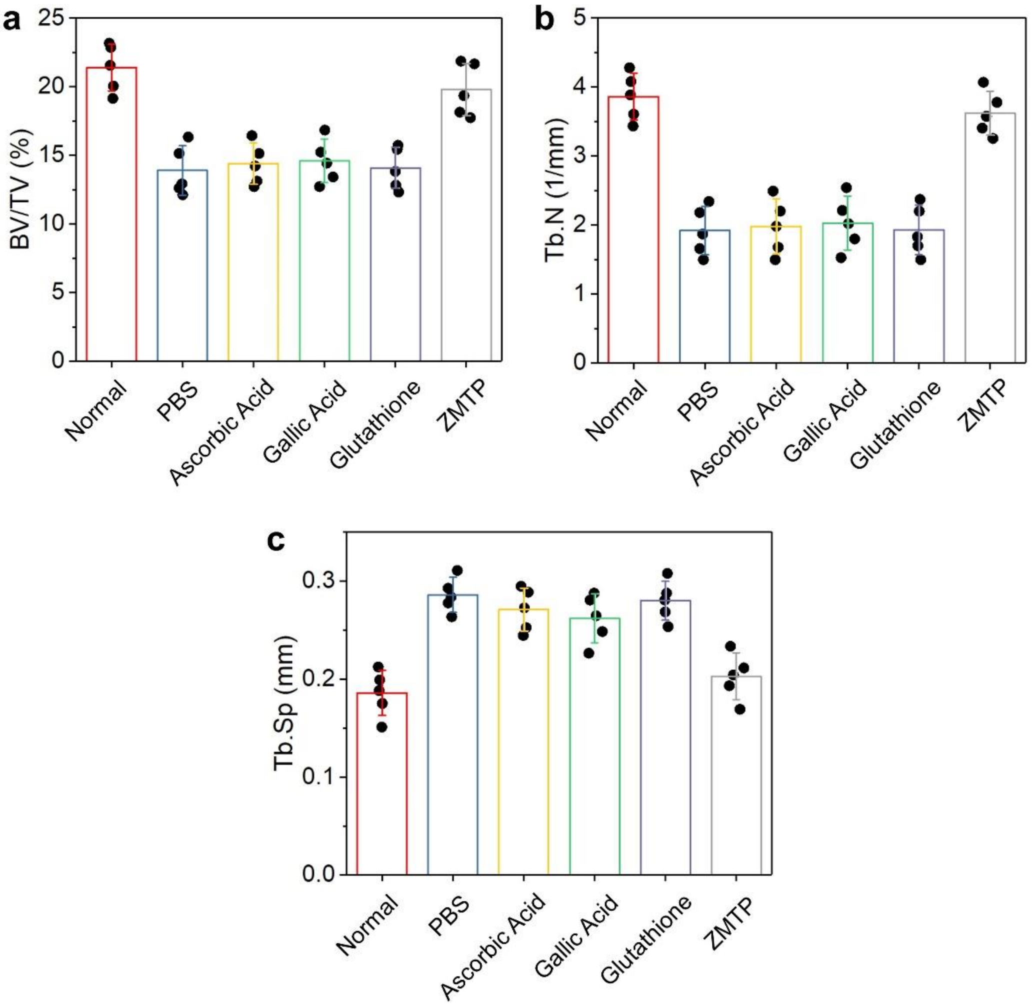

Supplementary Figure 29. Histomorphometric micro-CT analysis of fundamental parameters (BV/TV, Tb.N, and Tb.Sp) for right hind ankle joints of arthritic mice after treatment with ascorbic acid, gallic acid or glutathione (reduced) on day 36. The data for normal mice and arthritic mice in PBS and ZMTP groups are obtained from Figure 7d-7f for better comparison. Data are expressed as means ± SD (*N* = 5 biologically independent animals). Source data are provided as a Source Data file.

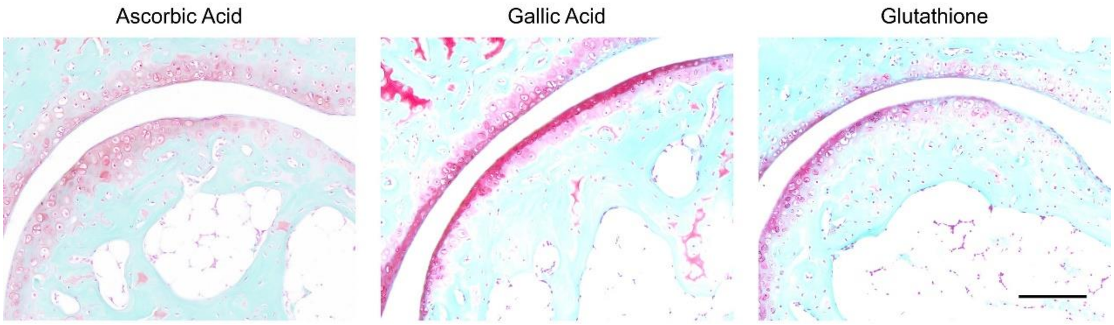

Supplementary Figure 30. Safranin-fixed green staining images of right hind ankle joints of arthritic mice after treatment with ascorbic acid, gallic acid or glutathione (reduced) on day 36, representative of five biological replicates from each group. Scale bar, 100 μm.

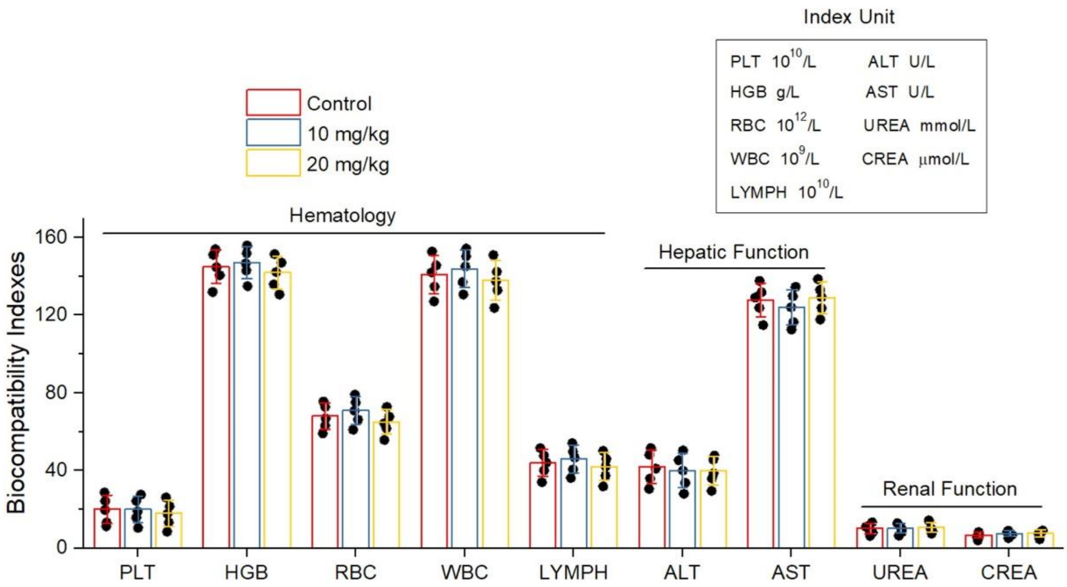

Supplementary Figure 31. Several key hematological, hepatic, and renal parameters of normal mice after treated with different doses of ZMTP nanosheets for 21 days. Data are expressed as means ± SD (N = 5 biologically independent animals). Source data are provided as a Source Data file.

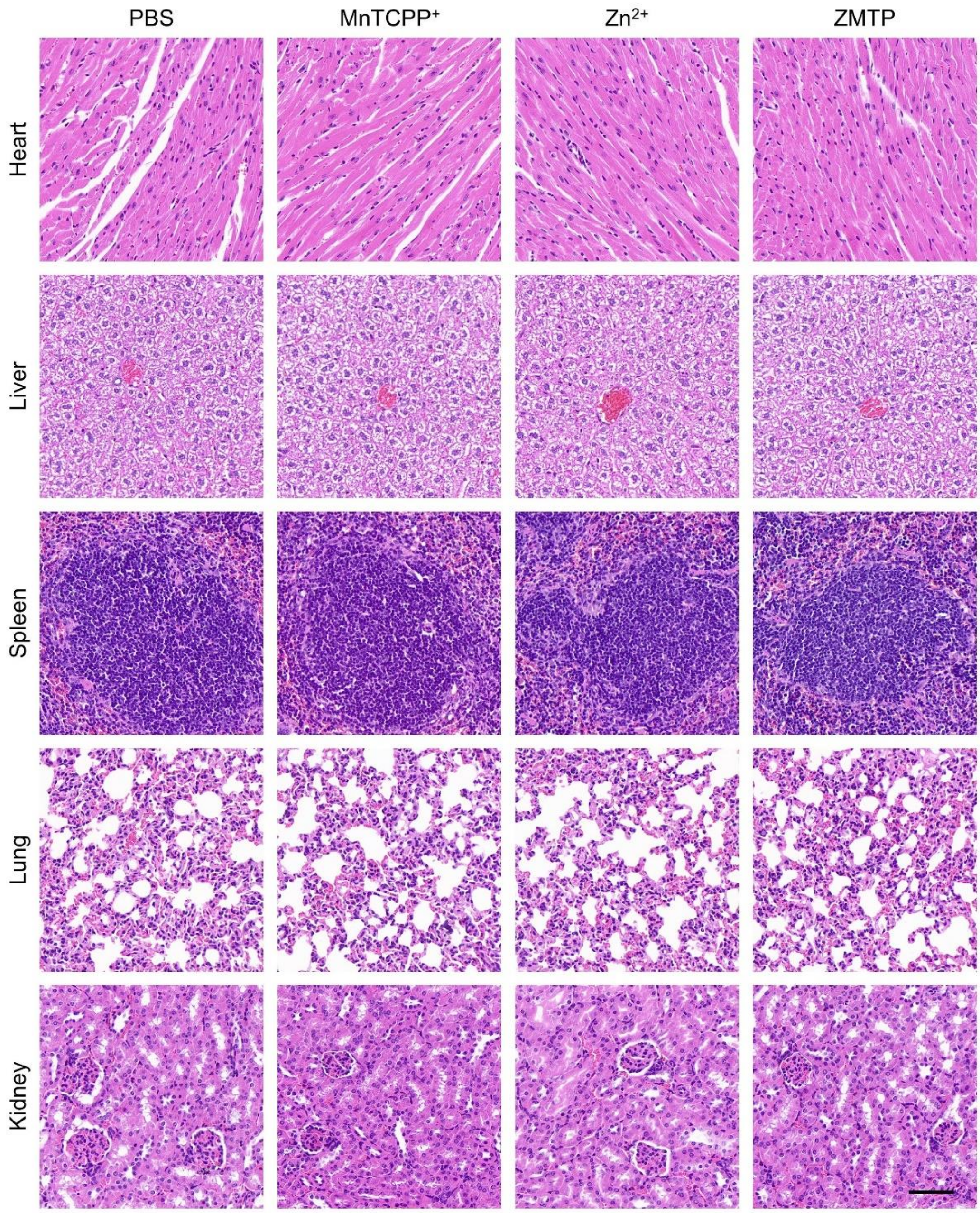

Supplementary Figure 32. H&E staining of hearts, livers, spleens, lungs, and kidneys harvested from normal mice after different treatments for 21 days. Scale bar, 50 μm. A representative image of five replicates from each experimental group is shown.

Supplementary Tables

Supplementary Table 1. Detailed chemical reactions for Mn-SOD or catalase-catalyzed ROS disproportionation.

Mn-SOD	$SOD\text{-}Mn^{III}(OH^{-}) + O_2^{\bullet-} + H^+ \rightarrow SOD\text{-}Mn^{II}(OH_2) + O_2$<br>$SOD\text{-}Mn^{II}(OH_2) + O_2^{\bullet-} + H^+ \rightarrow SOD\text{-}Mn^{III}(OH^{-}) + H_2O_2$
Catalase	$CAT\text{-}P\text{-}Fe^{III} + H_2O_2 \rightarrow CAT\text{-}P^{\bullet+}\text{-}Fe^{IV}{=}O + H_2O$<br>$CAT\text{-}P^{\bullet+}\text{-}Fe^{IV}{=}O + H_2O_2 \rightarrow CAT\text{-}P\text{-}Fe^{III} + H_2O + O_2$

Supplementary Table 2. Detailed chemical reactions during CV measurement in Figure 3b and Supplementary Figure 10 ($\iiint dxdydz = n$).

Mn^{2+}	Negative Scanning	$Mn^{3+} + e^{-} \rightarrow Mn^{2+}$
	Positive Scanning	$Mn^{2+} - e^{-} \rightarrow Mn^{3+}$
$MnTCPP^+$	Negative Scanning	$(H_2O)_2Mn^{III}TCPP^{3-} + e^{-} \rightarrow (H_2O)Mn^{II}TCPP^{4-} + H_2O$
	Positive Scanning	$(H_2O)Mn^{II}TCPP^{4-} + H_2O - e^{-} \rightarrow (H_2O)_2Mn^{III}TCPP^{3-}$
ZMTP	Negative Scanning	$\{Zn_2[(H_2O)(OH)Mn^{III}TCPP]\}_{(x,y,z)} + ne^{-} + nH^+ \rightarrow \{Zn_2[(H_2O)Mn^{II}TCPP]\}_{(x,y,z)} + nH_2O$
	Positive Scanning	$\{Zn_2[(H_2O)Mn^{II}TCPP]\}_{(x,y,z)} - ne^{-} + nH_2O \rightarrow \{Zn_2[(H_2O)(OH)Mn^{III}TCPP]\}_{(x,y,z)} + nH^+$

The electrochemical actions of $MnTCPP^+$ follow the dissociation of four benzoyloxy protons in aqueous solution.

Supplementary Table 3. Detailed chemical reactions during CA measurement in Figure 3c ($\iiint dxdydz = n$).

Anode	—
Cathode	$\{Zn_2[(H_2O)(OH)Mn^{III}TCPP]\}_{(x,y,z)} + ne^- + nH^+ \rightarrow \{Zn_2[(H_2O)Mn^{II}TCPP]\}_{(x,y,z)} + nH_2O$ (for the first period, while no reaction occurs in the subsequent periods)

Supplementary Table 4. Detailed chemical reactions during CA measurement in Figure 3d ($\iiint dxdydz = n$).

Anode	$\{Zn_2[(H_2O)Mn^{II}TCPP]\}_{(x,y,z)} - ne^- + nH_2O \rightarrow \{Zn_2[(H_2O)(OH)Mn^{III}TCPP]\}_{(x,y,z)} + nH^+$<br>$\{Zn_2[(H_2O)(OH)Mn^{III}TCPP]\}_{(x,y,z)} - 2ne^- \rightarrow \{Zn_2[(O)(OH)Mn^{V}TCPP]\}_{(x,y,z)} + 2nH^+$
Cathode	$\{Zn_2[(O)(OH)Mn^{V}TCPP]\}_{(x,y,z)} + 2ne^- + 2nH^+ \rightarrow \{Zn_2[(H_2O)(OH)Mn^{III}TCPP]\}_{(x,y,z)}$<br>$\{Zn_2[(H_2O)(OH)Mn^{III}TCPP]\}_{(x,y,z)} + ne^- + nH^+ \rightarrow \{Zn_2[(H_2O)Mn^{II}TCPP]\}_{(x,y,z)} + nH_2O$

Supplementary Table 5. Accumulated Zn element release from ZMTP nanosheets after CA measurements in Figure 3d and 3f, determined by ICP-OES.

Figure 3d (%)	Figure 3f (%)
0.483	1.961

Supplementary Table 6. Comparison of antioxidative activities among ZMTP and several common antioxidants (ascorbic acid, gallic acid, and reduced glutathione). Their concentrations required for inhibiting half amount of $O_2^{\bullet-}$ and H_2O_2 were determined.

Antioxidative Substance	ZMTP	Ascorbic Acid	Gallic Acid	Glutathione (reduced)
$C_{1/2}$ ($O_2^{\bullet-}$) (ppm)	0.74	8.81	8.52	768.4
$C_{1/2}$ (H_2O_2) (ppm)	1.73	4.68	5.83	375.6

Supplementary Table 7. Primer sequences used for RT-PCR quantification.[6]

Gene	Forward Sequencing Primer (5' to 3')	Reverse Sequencing Primer (5' to 3')
IL-6	GGAGCCCACCAAGAACGATA	ACCAGCATCAGTCCCAAGAA
IL-1β	ATGAAGGGCTGCTTCCAAAC	TCTCCACAGCCACAATGAGT
TNF-α	CTCATGCACCACCATCAAGG	ACCTGACCACTCTCCCTTTG
Arg-1	TGGCTTGCGAGACGTAGAC	GCTCAGGTGAATCGGCCTTT
IL-10	CTGGACAACATACTGCTAACCG	GGGCATCACTTCTACCAGGTAA
OPN	TTCTGAGGGACTAACTACGACCAT	CCAGAATCAGTCACTTTCACCG
OCN	ACCGTTGGTTGTTTGTCATTGC	GAGGTCAGTGGGGATGATAGAAG
ALP	GATAACGAGATGCCACCAGAGG	GTTCAGTGCGGTTCCAGACATAG
COL I	GCAAGAGGCGAGAGAGGTTT	GACCACGGGCACCATCTTTA
RUNX 2	ACACTGCCACCTCTGACTTCT	GGATGAAATGCTTGGGAACTGC
GAPDH	GGGTCCCAGCTTAGGTTCAT	CCAATACGGCCAAATCCGTT

Supplementary Table 8. $W_R/W_L(36)$ values of different experimental groups indicated in Supplementary Figure 26. For normal mice, $W_R/W_L(36) \approx 1$; For arthritic mice, $W_R/W_L(36) > 1$. The lower $W_R/W_L(36)$ value indicates the higher anti-inflammatory efficacy of treatment.

Treatment	PBS	Ascorbic Acid	Gallic Acid	Glutathione (reduced)	ZMTP
$W_R/W_L(36)$	1.63	1.55	1.54	1.60	1.17

Supplementary Table 9. Full names for the abbreviations in Supplementary Figure 31.

Abbreviation	Full Name
PLT	platelets
HGB	haemoglobin
RBC	red blood cells
WBC	white blood cells
LYMPH	lymphocytes
ALT	alanine aminotransferase
AST	aspartate aminotransferase
UREA	urea
CREA	creatinine

Supplementary References

1. Borgstahl, G. E. O., Parge, H. E., Hickey, M. J., Beyer, W. F., Hallewell, R. A. & Tainer, J. A. The structure of human mitochondrial manganese superoxide dismutase reveals a novel tetrameric interface of two 4-helix bundles. *Cell* **71**, 107-118 (1992).

2. Putnam, C. D., Arvai, A. S., Bourne, Y. & Tainer, J. A. Active and inhibited human catalase structures: ligand and NADPH binding and catalytic mechanism. *J. Mol. Biol.* **296**, 295-309 (2000).

3. Adam, S. M., Wijeratne, G. B., Rogler, P. J., Diaz, D. E., Quist, D. A., Liu, J. J. *et al.* Synthetic Fe/Cu complexes: Toward understanding heme-copper oxidase structure and function. *Chem. Rev.* **118**, 10840-11022 (2018).

4. Batinic-Haberle, I., Tovmasyan, A. & Spasojevic, I. An educational overview of the chemistry, biochemistry and therapeutic aspects of Mn porphyrins – From superoxide dismutation to H_2O_2-driven pathways. *Redox Biol.* **5**, 43-65 (2015).

5. Jin, N., Lahaye, D. E. & Groves, J. T. A "push-pull" mechanism for heterolytic O-O bond cleavage in hydroperoxo manganese porphyrins. *Inorg. Chem.* **49**, 11516-11524 (2010).

6. Yang, Y., Guo, L., Wang, Z., Liu, P., Liu, X., Ding, J. *et al.* Targeted silver nanoparticles for rheumatoid arthritis therapy via macrophage apoptosis and Re-polarization. *Biomaterials* **264**, 120390 (2021).
