# Supplementary material for: Construction of a two-dimensional artificial antioxidase for nanocatalytic rheumatoid arthritis treatment
Source: Nat Commun. 2022 Apr 13;13:1988. doi: 10.1038/s41467-022-29735-1 (PMC9008001; doi:10.1038/s41467-022-29735-1)
Supplement: Supplementary file 3 — Reporting Summary [file 41467_2022_29735_MOESM3_ESM.pdf]

nature portfolio

Corresponding author(s): Jianlin Shi

Last updated by author(s): Mar 6, 2022

Reporting Summary

Nature Portfolio wishes to improve the reproducibility of the work that we publish. This form provides structure for consistency and transparency in reporting. For further information on Nature Portfolio policies, see our Editorial Policies and the Editorial Policy Checklist.

Statistics

For all statistical analyses, confirm that the following items are present in the figure legend, table legend, main text, or Methods section.

n/a	Confirmed	
☐	☒	The exact sample size (n) for each experimental group/condition, given as a discrete number and unit of measurement
☐	☒	A statement on whether measurements were taken from distinct samples or whether the same sample was measured repeatedly
☒	☐	The statistical test(s) used AND whether they are one- or two-sided<br>*Only common tests should be described solely by name; describe more complex techniques in the Methods section.*
☒	☐	A description of all covariates tested
☒	☐	A description of any assumptions or corrections, such as tests of normality and adjustment for multiple comparisons
☐	☒	A full description of the statistical parameters including central tendency (e.g. means) or other basic estimates (e.g. regression coefficient) AND variation (e.g. standard deviation) or associated estimates of uncertainty (e.g. confidence intervals)
☒	☐	For null hypothesis testing, the test statistic (e.g. F, t, r) with confidence intervals, effect sizes, degrees of freedom and P value noted<br>*Give P values as exact values whenever suitable.*
☒	☐	For Bayesian analysis, information on the choice of priors and Markov chain Monte Carlo settings
☒	☐	For hierarchical and complex designs, identification of the appropriate level for tests and full reporting of outcomes
☒	☐	Estimates of effect sizes (e.g. Cohen's d, Pearson's r), indicating how they were calculated

Our web collection on statistics for biologists contains articles on many of the points above.

Software and code

Policy information about availability of computer code

Data collection	CytExpert (version 2.2)
Data analysis	FlowJo (version 10.0)

For manuscripts utilizing custom algorithms or software that are central to the research but not yet described in published literature, software must be made available to editors and reviewers. We strongly encourage code deposition in a community repository (e.g. GitHub). See the Nature Portfolio guidelines for submitting code & software for further information.

Data

Policy information about availability of data

All manuscripts must include a data availability statement. This statement should provide the following information, where applicable:
- Accession codes, unique identifiers, or web links for publicly available datasets
- A description of any restrictions on data availability
- For clinical datasets or third party data, please ensure that the statement adheres to our policy

The authors declare that all data needed to support the finding of this study are presented in the article and the Supplementary information. This study uses publicly available data from the Protein Data Bank (PDB) under accession codes: 1N0J (http://doi.org/10.2210/pdb1N0J/pdb), 1DGF (http://doi.org/10.2210/pdb1DGF/pdb). Any data related to this work are available from the corresponding authors upon reasonable request. A reporting summary for this article is available as a Supplementary Information file. Source data are provided with this paper.

Field-specific reporting

Please select the one below that is the best fit for your research. If you are not sure, read the appropriate sections before making your selection.

- [x] Life sciences
- [ ] Behavioural & social sciences
- [ ] Ecological, evolutionary & environmental sciences

For a reference copy of the document with all sections, see nature.com/documents/nr-reporting-summary-flat.pdf

Life sciences study design

All studies must disclose on these points even when the disclosure is negative.

Sample size	Sample sizes were consistent with other similar reports (e.g., Nat. Commun. 2021, 12, 3393).
Data exclusions	No data were excluded from the analyses.
Replication	All experiments were repeated at least three times with similar results.
Randomization	Cells or mice were randomly assigned to different groups before treatment. Other experiments are not related to this issue.
Blinding	CLSM images and optical images were collected by experienced operators who were blinded to the treatment groups.<br>CCK-8 assays, flow cytometry analysis, RT-PCR and alizarin red S staining were conducted by experienced operators who were blinded to the treatment groups.<br>CT analysis were conducted by experienced operators who were blinded to the treatment groups.<br>In histological assessments, tissues were analyzed by an experienced investigator who was blinded to the treatment groups.<br>For other experiments, the investigators were also blinded to group allocation during data collection.

Reporting for specific materials, systems and methods

We require information from authors about some types of materials, experimental systems and methods used in many studies. Here, indicate whether each material, system or method listed is relevant to your study. If you are not sure if a list item applies to your research, read the appropriate section before selecting a response.

Materials & experimental systems

n/a	Involved in the study	
x		Antibodies
	x	Eukaryotic cell lines
x		Palaeontology and archaeology
	x	Animals and other organisms
x		Human research participants
x		Clinical data
x		Dual use research of concern

Methods

n/a	Involved in the study	
x		ChIP-seq
	x	Flow cytometry
x		MRI-based neuroimaging

Eukaryotic cell lines

Policy information about cell lines

Cell line source(s)	Murine mononuclear macrophage cell line Raw 264.7 were kindly provided by Cell Bank/Stem Cell Bank, Chinese Academy of Sciences; murine bone mesenchymal stem cell line were kindly provided by ATCC.
Authentication	Cell lines were authenticated by Cell Bank/Stem Cell Bank, Chinese Academy of Sciences or ATCC using specific methods (Karyotyping, DNA barcoding, PCR assays with species-specific primers, etc.)
Mycoplasma contamination	Cell lines tested negative for mycoplasma.
Commonly misidentified lines (See ICLAC register)	No commonly misidentified cell lines were used.

Animals and other organisms

Policy information about studies involving animals; ARRIVE guidelines recommended for reporting animal research

Laboratory animals	Eighty female 8-week-old Balb/c mice were provided by Charles River, which were then housed under conditions of a light/dark cycle of 12 h, an ambient temperature of 25 ± 2 °C, and a humidity of 60 ± 10%.

Wild animals	This study did not involve wild animals
Field-collected samples	This study did not involve samples collected from the field.
Ethics oversight	The animal-related experiment procedures in this work were conducted according to the guidelines approved by the Animal Ethics Committee of Shanghai Tenth People's Hospital, Tongji University School of Medicine (SHDSYY-2020-Z0026/1)

Note that full information on the approval of the study protocol must also be provided in the manuscript.

Flow Cytometry

Plots

Confirm that:

- [x] The axis labels state the marker and fluorochrome used (e.g. CD4-FITC).
- [x] The axis scales are clearly visible. Include numbers along axes only for bottom left plot of group (a 'group' is an analysis of identical markers).
- [x] All plots are contour plots with outliers or pseudocolor plots.
- [x] A numerical value for number of cells or percentage (with statistics) is provided.

Methodology

Sample preparation	The treated cells were washed with PBS, collected by trypsinization (without EDTA), resuspended in cold PBS. Afterwards, obtained cells were stained with specific dyes according to the manufacturer's instruction and analyzed by flow cytometry.
Instrument	CytoFLEX flow cytometer (Beckman Coulter)
Software	Data collection: CytExpert version 2.2<br>Data analysis: FlowJo version 10.0
Cell population abundance	No cell sorting was performed.
Gating strategy	Live cells were selected by FSC and SSC analysis. Green fluorescence of DCF in cells was detected by FITC channel, while the green and red fluorescences of Annexin V-FITC and PI in cells was detected by FITC and PI channels, respectively.

- [x] Tick this box to confirm that a figure exemplifying the gating strategy is provided in the Supplementary Information.
